# In its very early phases, COVID-19 shifts the associations between alcohol consumption and psychological symptoms in young adults

**DOI:** 10.1192/j.eurpsy.2025.2450

**Published:** 2025-06-23

**Authors:** Karina Janson, Arun L.W. Bokde, Sylvane Desrivières, Hugh Garavan, Penny Gowland, Antoine Grigis, Andreas Heinz, Jean-Luc Martinot, Marie-Laure Paillère Martinot, Eric Artiges, Dimitri Papadopoulos Orfanos, Tomáš Paus, Luise Poustka, Michael N. Smolka, Nathalie E. Holz, Nilakshi Vaidya, Henrik Walter, Robert Whelan, Gunter Schumann, Herta Flor, Olaf Reis, Emanuel Schwarz, Tobias Banaschewski, Frauke Nees, IMAGEN Consortium, CoviDrug Consortium

**Affiliations:** 1 Institute of Medical Psychology and Medical Sociology, University Medical Center Schleswig-Holstein, Kiel University, Kiel, Germany; 2 Department of Child and Adolescent Psychiatry and Psychotherapy, Central Institute of Mental Health, Medical Faculty Mannheim, Heidelberg University, Mannheim, Germany; 3 Discipline of Psychiatry, School of Medicine and Trinity College Institute of Neuroscience, Trinity College Dublin, Dublin, Ireland; 4 Centre for Population Neuroscience and Precision Medicine (PONS), Institute of Psychiatry, Psychology & Neuroscience, SGDP Centre, King’s College London, UK; 5 Departments of Psychiatry and Psychology, University of Vermont, Burlington, VT, USA; 6 Sir Peter Mansfield Imaginge Centre School of Physics and Astronomy, University of Nottingham, Nottingham, UK; 7 NeuroSpin, CEA, Université Paris-Saclay, Gif-sur-Yvette, France; 8 Department of Psychiatry and Psychotherapy CCM, Charité – Universitätsmedizin Berlin, corporate member of Freie Universität Berlin, Humboldt-Universität zu Berlin, and Berlin Institute of Health, Berlin, Germany; 9 Institut National de la Santé et de la Recherce Médicale, INSERM U A10 “Trajectoires développementales & psychiatrie,” University Paris-Saclay, Ecole Normale Supérieure Paris-Saclay, CNRS, Centre Borelli, Gif-sur-Yvette, France; 10 Department of Child and Adolescent Psychiatry, Sorbonne Université, Pitié-Salpêtrière Hospital, Paris, France; 11 Psychiatry Department, EPS Barthélémy Durand, Etampes, France; 12 Department of Psychiatry, Faculty of Medicine and Centre Hospitalier Universitaire Sainte-Justine, University of Montreal, Montreal, Quebec, Canada; 13 Departments of Psychiatry and Psychology, University of Toronto, Toronto, ON, Canada; 14 Department of Child and Adolescent Psychiatry, Center for Psychosocial Medicine, University Hospital Heidelberg, Heidelberg, Germany; 15 Department of Psychiatry and Neuroimaging Center, Technische Universität Dresden, Dresden, Germany; 16 German Center for Mental Health (DZPG), Partner Site Mannheim-Heidelberg-Ulm, Germany; 17 Centre for Population Neuroscience and Stratified Medicine (PONS), Department of Psychiatry and Neuroscience, Charité Universitätsmedizin Berlin, Berlin, Germany; 18 School of Psychology and Global Brain Health Institute, Trinity College Dublin, Dublin, Ireland; 19 Centre for Population Neuroscience and Stratified Medicine (PONS), Institute for Science and Technology of Brain-inspired Intelligence (ISTBI), Fudan Universität, Shanghai, China; 20 Institute of Cognitive and Clinical Neuroscience, Central Institute of Mental Health, Medical Faculty Mannheim, Heidelberg University, Mannheim, Germany; 21 Department of Psychology, School of Social Sciences, University of Mannheim, Mannheim, Germany; 22 Department of Child and Adolescent Psychiatry, University Medical Centre Rostock, Rostock, Germany; 23 German Center for Child and Adolescent Health (DZKJ), Rostock, Germany; 24 Department of Psychiatry and Psychotherapy, Central Institute of Mental Health, Medical Faculty Mannheim, Heidelberg University, Mannheim, Germany; 25 Hector Institute for Artificial Intelligence in Psychiatry, Central Institute of Mental Health, Medical Faculty Mannheim, Heidelberg University, Mannheim, Germany

**Keywords:** alcohol use, anxiety, Covid-19, depression, mental health, psychological symptoms

## Abstract

**Background:**

The COVID-19 pandemic has impacted various aspects of daily life, leading to increased psychological symptoms and changes in alcohol use, yet little is known about their specific interactions, particularly early stages during the pandemic. We examined the relationship between psychological symptoms and alcohol-related behaviors associated with COVID-19, and determined whether associations shifted already early during the pandemic and whether changes in psychological symptoms from the pre- to during COVID-19 impacted changes in alcohol consumption.

**Methods:**

Participants were young adults from a longitudinal cohort (N=435, age: 22–25) from two time points. We applied paired samples t-tests, correlation analyses, SHapley Additive exPlanations, and classification models to examine the multiple associations between psychological symptoms and alcohol use directly pre- and early during COVID-19.

**Results:**

We found significant associations between psychological symptoms and alcohol use pre- compared to during COVID-19. Anxiety was the strongest factor influencing alcohol use pre-pandemic, depression had the greatest impact during COVID-19. Changes in anxiety from pre- to during COVID-19 were the main factor associated with an increase in alcohol use, while changes in depression appeared to be most predictive for a decrease/persistence in alcohol use.

**Conclusion:**

These findings suggest a shift in the association between psychological symptoms and alcohol use following COVID-19, as well as a differential impact of psychological symptoms, depending on their changes related to the pandemic. Changes in anxiety may contribute to riskier alcohol use behaviors following the pandemic, while depression appears to be one of the most critical factors influencing alcohol use during such crisis situations.

## Introduction

The COVID-19 pandemic has brought about significant changes in various aspects of daily life, leading to an increase in psychological symptoms and a decline in mental health, as well as shifts in alcohol use behaviors (e.g., [1–3]). Evidence consistently highlighted a rise in psychological distress, emphasizing the negative impact of the pandemic on well-being and mental health [4].

In contrast, the impact of the pandemic on alcohol consumption has been less clear. It was shown that alcohol use increased (e.g., [5, 6]), also depending on subgroup-specific aspects such as socio-demographics as well as age (e.g., [5]) and with changes in social drinking habits [6], or decreased, potentially due to restricted availability and financial limitations during the pandemic [7]. Some studies reported no significant changes in alcohol consumption [8], while others observed a decrease [9]. Given that studies have shown that psychological distress often co-occurs with problematic alcohol consumption [10, 11] and that social isolation and stress are key factors in disordered drinking [12], there may be a relationship between changes in well-being from pre-pandemic to during the pandemic and shifts in alcohol use behaviors.

This may have already manifested at the very onset of the pandemic, when changes in daily life were most pronounced. Even though the pandemic is now years in the past, its relevance for clinical practice remains evident, particularly the importance of understanding and considering early processing and reactions. These early responses could serve as significant triggers for later reactions and developments, potentially helping to identify subgroups that, due to these initial responses, require different treatment approaches. Such experiences and reactions may persist (e.g., in the form of a generally altered coping style in challenging situations), even if they do not appear to have an immediate impact on the current situation.

Substance use, typically associated with social reinforcement, may shift during crises toward coping mechanisms driven by negative internal states [13, 14]. While the link between psychological symptoms and alcohol consumption is well-established [15, 16], the intricate interplay of these factors may evolve due to the unique stressors and uncertainties introduced by the pandemic. This dynamic could have been particularly pronounced during the early phase of the pandemic, when challenges and restrictions in daily life were at their peak. Previous studies that have looked at these early periods of the COVID-19 pandemic have found an increase in depression and anxiety symptoms, sleep, and smoking, as well as increased drinking, to cope with worries [17–19].

Examining patterns from this period that extend beyond the well-known core aspects of depression and anxiety to include broader psychological symptoms, as well as their specific interaction with alcohol use and the comorbid presence of elevated symptoms within these spectrums, could offer valuable insights into the trajectories of these behaviors during later stages of the pandemic and the post-pandemic period. Furthermore, it is essential to examine data from an age-relatively homogeneous sample to minimize potential age-related experiential effects. Early adulthood is particularly important in the context of prevention and early intervention.

In the present study, we aimed to investigate the associations between psychological symptoms and alcohol consumption both several years prior to and during the early phase of the COVID-19 pandemic, using a longitudinal approach in individuals in early adulthood. Through the application of group and correlation analyses as well as machine learning, we specifically sought to address the following research questions: 1. Are there differences in the levels of alcohol consumption and psychological symptoms between the pre-pandemic period and the early phase of the COVID-19 pandemic? 2. Do the levels of alcohol consumption and psychological symptoms change from the pre-pandemic period to the early phase of the pandemic, and how are these changes related to the association between psychological symptoms and alcohol consumption?

## Methods

### Data source

**Figure 1.**
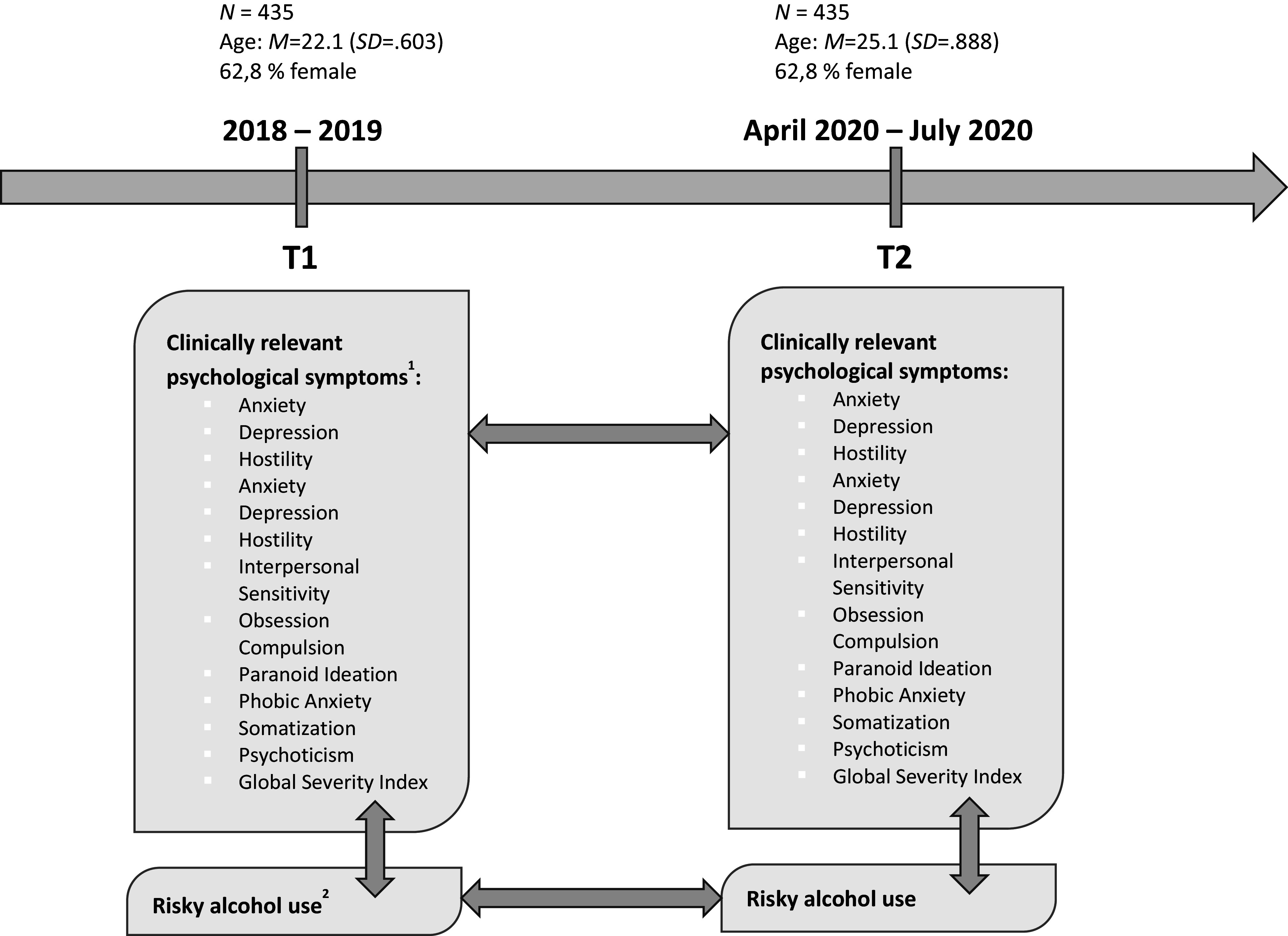
Data assessment before (T1, FU3 assessment in Imaging Genetics (IMAGEN)) and during COVID-19 (T2); ^1^using the Brief Symptom Inventory (BSI), using the Alcohol Use Disorders Identification Test (AUDIT, total score).

In the present study, we investigated a sample of healthy young adults (N = 435, age range 22–25 years) from the longitudinal Imaging Genetics (IMAGEN) study [20]) for whom data pre- and during COVID-19 exist (Figure 1). Young adults were recruited in Germany, Ireland, France, and the United Kingdom through school visits, flyers, and registration offices. Exclusion criteria were serious medical conditions, pregnancy, and previous head trauma with unconsciousness. The COVID assessments were performed in addition to the general longitudinal baseline and follow-up timeline, and participation was voluntary, with only a subset of individuals choosing to take part in this assessment. We included in our analyses only participants with complete data for all required variables (BSI, demographics, and AUDIT) at both measurement timepoints.

### Ethics statement

The authors assert that all procedures contributing to this work comply with the ethical standards of the relevant national and institutional committees on human experimentation and with the Helsinki Declaration of 1975, as revised in 2008. All procedures involving human subjects were approved by the local ethics committees of the recruitment centers. All participants and their guardians provided written or online informed consent prior to participation.

### Measures

#### Assessment of psychological symptoms

We used the Brief Symptom Inventory (BSI), which covers nine symptom dimensions (Table 1): somatization, obsession-compulsion, interpersonal sensitivity, depression, anxiety, hostility, phobic anxiety, paranoid ideation and psychoticism, and the global severity index, which represents the sum of those dimensions [21].

**Table 1.** Means, standard deviations, and Cronbach’s alphas for study variables and comparison of mental health pre-pandemic to during the pandemic

	Pre-pandemic		Pandemic		Time comparison		
	M (SD)	Cronbach’s Alpha	M (SD)	Cronbach’s Alpha	t	p	d
*BSI scales*							
Anxiety	1.37 (1.607)	.823	2.52 (3.314)	.786	−7.98	.000	−.287
Depression	1.76 (1.887)	.876	3.59 (4.126)	.839	−10.77	.000	−.419
Hostility	1.24 (1.238)	.721	2.26 (2.774)	.786	−8.36	.000	−.305
Interpersonal sensitivity	1.25 (1.352)	.782	2.60 (3.231)	.820	−10.11	.000	−.387
Obsession-compulsion	2.32 (1.837)	.812	4.48 (4.109)	.806	−12.24	.000	−.487
Paranoid ideation	1.15 (1.428)	.774	1.92 (2.957)	.789	−6.29	.000	−.206
Phobic anxiety	0.58 (1.055)	.750	1.63 (2.542)	.664	−9.21	.000	−.348
Somatization	1.16 (1.643)	.808	1.87 (2.969)	.770	−5.81	.000	−.280
Psychoticism	0.85 (1.102)	.685	1.65 (2.516)	.687	−7.49	.000	−.264
Global severity index	12.86 (10.849)	.957	23.69 (22.101)	.947	−12.5	.000	−.506
*AUDIT scales*							
Total score	6.02 (4.234)	.787	5.09 (4.019)	.745	5.54	.000	.363

#### Assessment of alcohol consumption

For the assessment of alcohol consumption, we used the Alcohol Use Disorders Identification Test (AUDIT) score for our analyses [22]. The AUDIT Alcohol Assessment is a globally recognized tool designed to evaluate alcohol consumption habits, providing valuable insights into individuals’ drinking patterns and associated risks [22, 23]. It serves as a standard for identifying different levels of alcohol use and the potential for dependency. The AUDIT score (Table 1) comprises nine items that collectively represent an individual’s alcohol consumption behavior. This scoring system allows for a comprehensive evaluation of alcohol use patterns and related behaviors, which provided a valid indicator concerning our research question and, in relation to previous studies, specifically of the IMAGEN cohort, to be able to link the results with the existing knowledge from the studies.

#### Statistical analysis

The present study aimed to explore the relationship between psychological symptoms and alcohol consumption before and during the COVID-19 pandemic, including the predictive impact of psychological symptoms (using the BSI scales) on alcohol use (using the AUDIT total score). To achieve this, we have employed a series of detailed analyses using R Studio’s *ggstatsplot* library. We performed correlation analyses to examine the associations between the BSI scales and AUDIT scores separately for the pre-COVID-19 and during COVID-19 periods. We conducted paired samples t-tests to assess whether BSI and AUDIT scores have significantly changed pre- versus during COVID-19. We calculated difference scores of BSI and AUDIT score levels pre- versus during COVID-19 for each participant. These difference scores were then used in further analyses to examine if and how the changes in psychological symptoms and alcohol consumption are associated (for distribution plots for both BSI scales and AUDIT scores, see Supplementary Material 1–3). These analyses included correlation analyses as well as SHapley Additive exPlanations (SHAP) and classification models. This analysis was conducted in two contexts: first, predicting AUDIT scores during COVID-19 using pre-COVID-19 BSI subscales, and second, predicting changes in AUDIT scores (pre- to during-COVID-19) using changes in BSI subscales. Model performance was evaluated using accuracy, precision, recall, and F1 scores. The SHAP values indicate the relative importance of each feature (BSI scales) in predicting alcohol use (AUDIT scores), both during COVID-19 as well as for the change from pre- to during COVID-19. For the latter one, participants were grouped in alcohol use into three categories, based on their difference scores: increased consumption, decreased consumption, and persistent consumption. SHAP values were used to interpret the impact of each psychological symptom on the likelihood of a participant falling into one of the three alcohol consumption groups (increase, decrease, and persistent). We employed the brute force method to determine the most predictive subset of psychological symptom changes (subscales) for forecasting the level of alcohol use (AUDIT total score) during COVID-19 and the changes from pre- to during COVID-19. This approach systematically evaluates all possible subsets of predictors to determine which combination yields the best model performance. Finally, to also take into account the changes in psychological symptoms pre- versus during COVID-19, we performed a classification model using the difference values of psychological symptoms as predictors and the categorized changes in alcohol consumption (increase, decrease, and persistent) as the target variable. Throughout the analyses, we controlled for covariates including age and center, and, for the change-related associations, also the pre-COVID-19 levels, to account for potential confounding variables and effects. Throughout the analyses, covariates including age, center, and pre-COVID-19 AUDIT levels were included to control for potential confounding effects. A significance threshold of p < .05 was applied for all statistical tests, with corrections for multiple comparisons conducted using the Benjamini-Hochberg procedure. For all significant findings, effect sizes (e.g., Cohen’s d, r) were calculated to contextualize the magnitude of the effects.

## Results

### Associations between alcohol consumption and psychological symptoms pre-COVID-19 and during COVID-19

**Figure 2.**
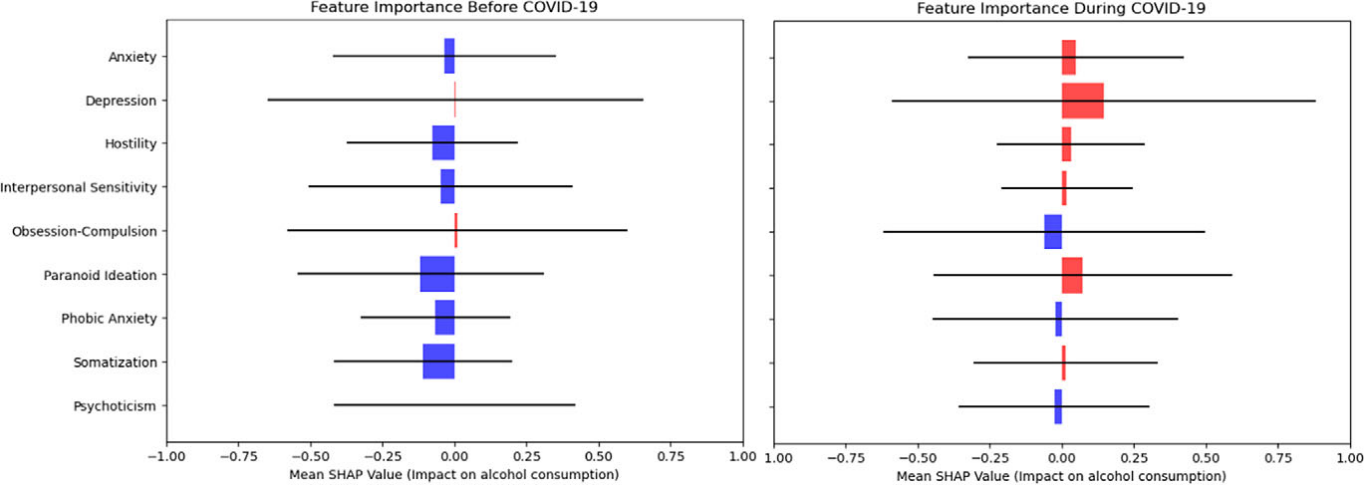
SHapley Additive exPlanations (SHAP) values for psychological symptoms predicting alcohol use pre- and during COVID-19. The SHAP values represent the relative importance of each psychological symptom (BSI subscale) in predicting AUDIT scores. Positive SHAP values (in red) indicate that higher symptom levels increase the likelihood of higher alcohol use (AUDIT scores), while negative SHAP values (in blue) indicate the opposite effect. Horizontal bars reflect the variability of SHAP values across participants, providing insight into the consistency of each symptom’s contribution to model predictions. The mean SHAP values, shown as points, summarize the overall contribution of each symptom. It is important to note that SHAP values are not statistical confidence intervals but rather describe the magnitude of feature importance in the model.

We found a diverse set of significant correlations between several psychological symptoms and alcohol consumption both pre- and during COVID-19. While BSI anxiety (pre: *r* = 0.102, *p* = 0.036; during: *r* = 0.146, *p* = 0.003) and psychoticism (pre: *r* = 0.112, *p* = 0.022; during: *r* = 0.115, *p* = 0.019) were significantly positively correlated with alcohol use both pre-COVID-19 and during COVID-19, in addition, BSI depression (*r* = 0.192, *p* < 0.001), interpersonal sensitivity (*r* = 0.146, *p* = 0.003), obsession-compulsion (*r* = 0.134, *p* = 0.006), paranoid ideation (*r* = 0.141, *p* = 0.004), and the global severity index (*r* = 0.166, *p* < 0.001) were all significantly positively correlated with AUDIT scores. The SHAP analyses also showed varied impact of and distinct importance among psychological symptoms on alcohol use pre-COVID-19 and during COVID-19 periods (Figure 2). For example, while pre-COVID-19, depression had a minimal influence on alcohol use (SHAP value of 0.00040), during COVID-19, depression emerged as the most influential factor, with a mean SHAP value of 0.1463.

### Changes of levels of alcohol consumption and psychological symptoms from pre-COVID-19 to during COVID-19 and their associations

**Figure 3.**
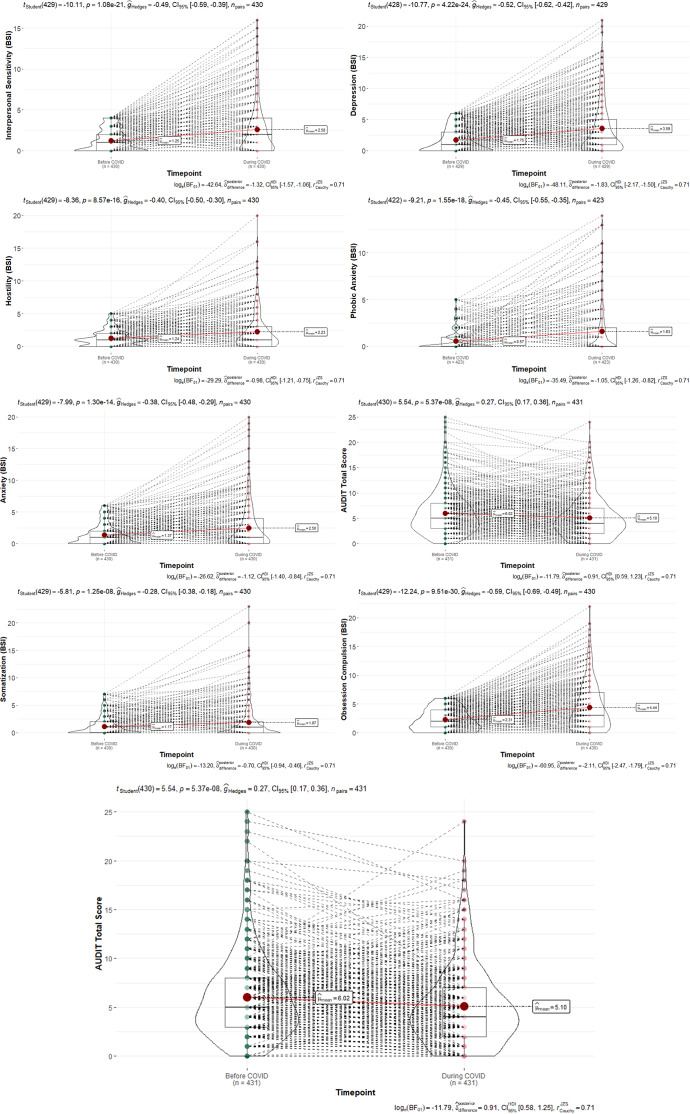
Changes in BSI scales and AUDIT scores from pre- to during COVID-19.

For pre-compared during COVID-19, we found significant differences for both the AUDIT scores and the BSI scales (Figure 3). Participants showed significantly higher levels in all BSI scales and significantly lower levels in the AUDIT total score during versus pre-COVID-19 (Table 2).

**Table 2.** Changes from pre-pandemic to during the pandemic grouped in clusters

	Increase n (%)	Decrease n (%)	Persistent n (%)
*BSI scales*			
Anxiety	229 (52.6%)	85 (19.5%)	121 (27.8%)
Depression	291 (66.9%)	55 (12.6%)	89 (20.5%)
Hostility	238 (54.7%)	82 (18.9%)	115 (26.4%)
Interpersonal sensitivity	242 (55.6%)	51 (11.7%)	142 (32.6%)
Obsession-compulsion	334 (76.8%)	50 (11.5%)	51 (11.7%)
Paranoid ideation	193 (44.4%)	92 (21.1%)	150 (34.5%)
Phobic anxiety	183 (42.1%)	51 (11.7%)	201 (46.2%)
Somatization	205 (47.1%)	77 (17.7%)	153 (35.2%)
Psychoticism	188 (43.2%)	75 (17.2%)	172 (39.5%)
Global severity index	305 (70.1%)	95 (21.8%)	18 (4.1%)
*AUDIT scales*			
Total score	114 (26.2%)	235 (54.0%)	82 (18.9%)

**Figure 4.**
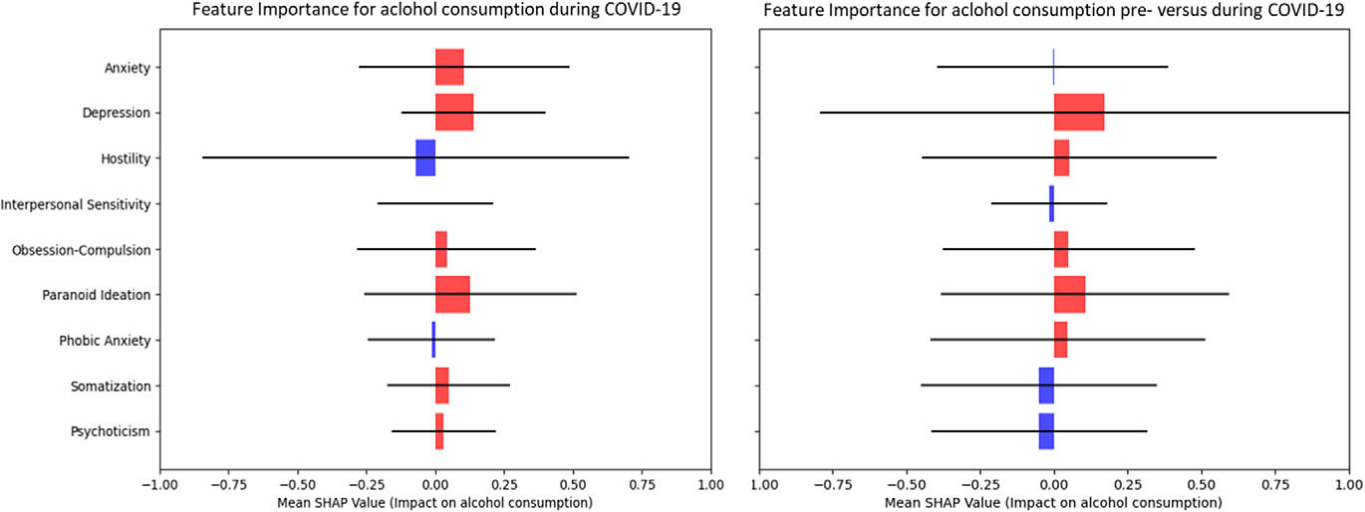
Left: SHAP models for the relative impact of difference scores of psychological symptoms (BSI scales) pre- versus during COVID-19 for alcohol consumption (AUDIT scores). Right: SHAP models for the relative impact of difference scores of psychological symptoms (BSI scales) pre- versus during COVID-19 for difference scores in alcohol consumption (AUDIT scores).

SHAP models showed that changes in depression and paranoid ideation were the strongest factors for alcohol use during COVID-19, and changes in anxiety, depression, and paranoid ideation were most strongly associated with changes in alcohol use pre- versus during COVID-19 (Figure 4).

**Figure 5.**
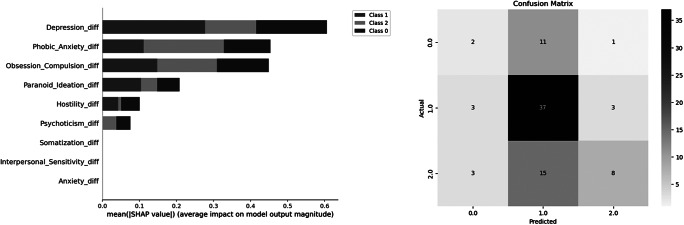
Left: The classification report for the prediction of three alcohol consumption classes pre- versus during COVID-19 by changes in psychological symptoms pre- versus during COVID-19. Middle: The confusion matrix, which illustrates that the model had the highest recall for class 1, suggesting that it was particularly effective in identifying this group. Right: Overview on the number of participants in each of the clustered groups by psychological symptom scales and alcohol use. Note: class 0 = no change in alcohol use pre- versus during COVID-19, class 1 = decrease in alcohol consumption pre- versus during COVID-19, and class 2 = increase in alcohol consumption pre- versus during COVID-19.

Moreover, while depression was mainly predictive for a decrease in alcohol use, anxiety served as the main factor for an increase in alcohol consumption pre-compared to during COVID-19 (Figure 5). The classification model achieved an overall accuracy of 56.63%, which, while modest, exceeds the chance level of 50% and reflects the complexity of the relationship between psychological symptoms and alcohol use. The detailed classification report showed varying precision, recall, and F1 scores for different groups, highlighting the model’s strengths and weaknesses in predicting categories of alcohol consumption, with the following performance metrics: a precision of 0.25 for class 0 (no change in AUDIT scores), 0.59 for class 1 (decrease in AUDIT scores), and 0.67 for class 2 (increase in AUDIT scores), with corresponding recall values of 0.14, 0.86, and 0.31, respectively (Figure 5). The weighted average F1 score was 0.52, reflecting the model’s overall balanced performance across different classes. Results of cross-validation and interaction plots are presented in Supplementary Material 8–11.

## Discussion

Studies have demonstrated that the COVID-19 pandemic has led to a significant increase in psychological symptoms and changes in alcohol consumption patterns (e.g., [1–3]). However, there remains limited understanding of the interactions between these factors during the pandemic and how they differ from the pre-pandemic period. In this study, we used longitudinal data from a large cohort study and applied group-based analyses, correlation techniques, and machine learning approaches (SHAP and classification models) to investigate the associations between psychological symptoms and alcohol use in individuals during early adulthood, comparing the pre-pandemic to the early phase of COVID-19.

Existing models of alcohol consumption have highlighted factors such as stress, social reinforcement, and psychological symptoms like anxiety and depression as key influences on drinking patterns [24, 25]). However, global crises like the COVID-19 pandemic may have disrupted these dynamics. The pandemic has been perceived as a stressful and negative life event [26], altered the social environment and associated factors [27], and resulted in an increase in psychological symptoms, including anxiety and depression [28].

Consistent with previous studies (also, e.g., [1–3, 9]), we observed significantly higher levels of psychological symptoms and lower levels of alcohol consumption when comparing the pre-pandemic periods to the early phase of COVID-19. The rise in psychological symptoms likely reflects reactions to the numerous challenges brought about by the pandemic. Although some previous studies have reported an increase in alcohol use [9, 29], the overall decline in alcohol consumption observed in our study aligns with findings from other studies [3, 30]. This reduction may be attributed to social contact restrictions and limited access to alcohol during lockdowns in the early period of the pandemic [31], when our COVID-19 data were collected.

The age range of our sample (young adulthood) is also a relevant factor, given this period’s heightened sensitivity to environmental and health influences and the critical role of social factors and contexts, including those related to alcohol consumption.

Moreover, previous findings reporting increases, decreases, or no changes in alcohol use when comparing pre- and during COVID-19 periods highlight substantial individual variation in alcohol use. This suggests the presence of underlying effects that may not be immediately apparent, warranting further investigation to better understand alcohol use behaviors during the pandemic.

When classifying individuals into groups based on increased, decreased, or stable alcohol consumption from pre- to during COVID-19, distinct psychological symptoms emerged as predictors of these changes. Anxiety was the primary factor associated with increases in alcohol use, while depression was more strongly linked to decreases or stable patterns of alcohol consumption.

A recent study using data from the same sample, along with data from clinically depressed individuals, identified varying trajectories of depression during the pandemic [3]. These findings underscore the importance of considering potentially distinct associations when examining risk and resilience factors during, and now also following, the pandemic.

Such distinct associations were also observed in our study. Coping behaviors may have intensified during the pandemic, potentially shifting the motives for alcohol use – for example, from social motives driven by peer relationships to internal reinforces such as feelings and thoughts [13, 14].

Prior to the pandemic, we observed significant positive correlations between alcohol consumption and psychological symptoms of anxiety and psychoticism. During the pandemic, additional significant correlations emerged, including those with depression, obsessive-compulsive symptoms, and interpersonal sensitivity. While depression had a minimal influence on alcohol consumption before COVID-19, it became the most influential factor during the pandemic.

In our analyses, we utilized the total score of the AUDIT questionnaire as a measure of alcohol-related behaviors (according to [32]). While the global AUDIT score is primarily a tool for assessing the risk of alcohol use disorder, it also reflects a comprehensive information that integrates both consumption patterns and alcohol-related consequences. This approach aligns with our study’s objectives, as it allows for a holistic understanding of changes in alcohol use behaviors (see also [22]) and their associations with psychological symptoms. In the manuscript, we therefore speak about alcohol use behaviors when referring to the score.

While these findings underscore the critical role of psychological symptoms in influencing alcohol use behaviors during crises [33] and suggest that the specific symptoms influencing alcohol use may shift during challenging periods, one could also argue that these differences might be related to changes in the variance of each variable pre- compared to during COVID-19. However, these differences were not statistically significant (see Supplementary Information). Additionally, the pre-COVID-19 levels could have affected the change-related associations [3], which is why we included them as covariates in our analyses. Therefore, the observed effects should primarily be attributed to the pandemic itself, along with its numerous disruptions and consequences. To further investigate the relationship between psychological symptoms and alcohol use, we examined whether baseline anxiety predicted later alcohol use while controlling for baseline AUDIT scores. The results did not indicate a significant longitudinal association, suggesting that baseline anxiety alone does not drive changes in alcohol-related behaviors beyond the initial alcohol use level (see Supplementary Information). This finding underscores the complex interplay between psychological distress and drinking behaviors, indicating that additional factors, such as coping mechanisms, social influences, or personality traits, may contribute more prominently to changes in alcohol use over time.

Addictive behaviors have long been understood through multifaceted models that encompass genetic, environmental, and psychological factors [34]. This approach is crucial for understanding the changes and patterns we observed in the associations between psychological symptoms and alcohol consumption before and during COVID-19, as other factors may have influenced these dynamics. Future research should therefore incorporate more psychosocial and environmental data, when available, to further explore these interactions during such critical periods in a medical crisis, helping to inform strategies for potential future scenarios. Moreover, future studies should continue to investigate these interactions during later stages of the pandemic and in the post-pandemic period.

Our study has several limitations. The reliance on self-reported data, while common in epidemiological research, may introduce biases such as recall or social desirability bias. Future research could incorporate physiological markers to complement self-reported behaviors. Additionally, the generalizability of our findings may be limited to specific demographic or cultural contexts, underscoring the need for cross-cultural studies to better understand global patterns of substance use during crises. Another limitation pertains to participant retention and representativeness. The COVID assessments were an additional voluntary measure within the longitudinal IMAGEN framework, leading to a reduced sample size compared to the original cohort. While we included only participants with complete data at both COVID timepoints, this selection process may have introduced biases, which should be kept in mind regarding the generalizability of our findings. Finally, one limitation pertains to the use of the AUDIT score. The AUDIT is widely used to assess alcohol-related behaviors [22, 35]; however, we also want to note that it is primarily designed to identify individuals at risk for alcohol use disorder rather than to directly measure alcohol consumption levels. Given that the total AUDIT score integrates both consumption-related and consequence-related factors, we decided to use this score as our indicator, as it fits with our hypotheses and with previous studies as an important aspect for comparisons, but we also want to specify that the categorization of alcohol use changes (increased, decreased, and persistent use) done in the present study is based on a broader risk framework rather than solely on consumption frequency. For future studies, it could be interesting to consider also alternative measures that more precisely capture consumption behaviors to reflect other issues of alcohol use.

## Conclusion

In conclusion, our study explored the multiple associations between psychological symptoms and alcohol use both a few years before and very early during the COVID-19 pandemic in an age-homogeneous sample of young adults. Our findings suggest a change in these associations, with depression appearing as the main factor linked to a decrease in alcohol use during the pandemic, but not before, and anxiety being the primary factor associated with an increase in alcohol use before versus during COVID-19. These results offer valuable insights for prevention and intervention strategies. Future studies should further refine and complement existing interaction models to understand how extreme environmental stressors interact with pre-existing vulnerabilities to influence alcohol use behaviors [24, 25]. Moreover, future research could examine the impact of environmental changes and challenges during later periods of the pandemic and in the post-pandemic. Here, one study on adult twin pairs provided some first information by controlling for genetic and shared environmental factors [18]. Still, integrating additional contextual factors, when available, could help enrich strategies for effective public health responses.

## Supporting information

10.1192/j.eurpsy.2025.2450.sm001 Janson et al. supplementary material Janson et al. supplementary material

## Data Availability

The datasets used and/or analyzed during the current study are available from the corresponding author on reasonable request.

## Supplementary material

The supplementary material for this article can be found at http://doi.org/10.1192/j.eurpsy.2025.2450.
