# Supplementary material for: In its very early phases, COVID-19 shifts the associations between alcohol consumption and psychological symptoms in young adults
Source: Eur Psychiatry. 2025 Jun 23;68(1):e77. doi: 10.1192/j.eurpsy.2025.2450 (PMC12188332; doi:10.1192/j.eurpsy.2025.2450)
Supplement: Janson et al. supplementary material [file S0924933825024502sup001.docx]

**SUPPLEMENT**

**
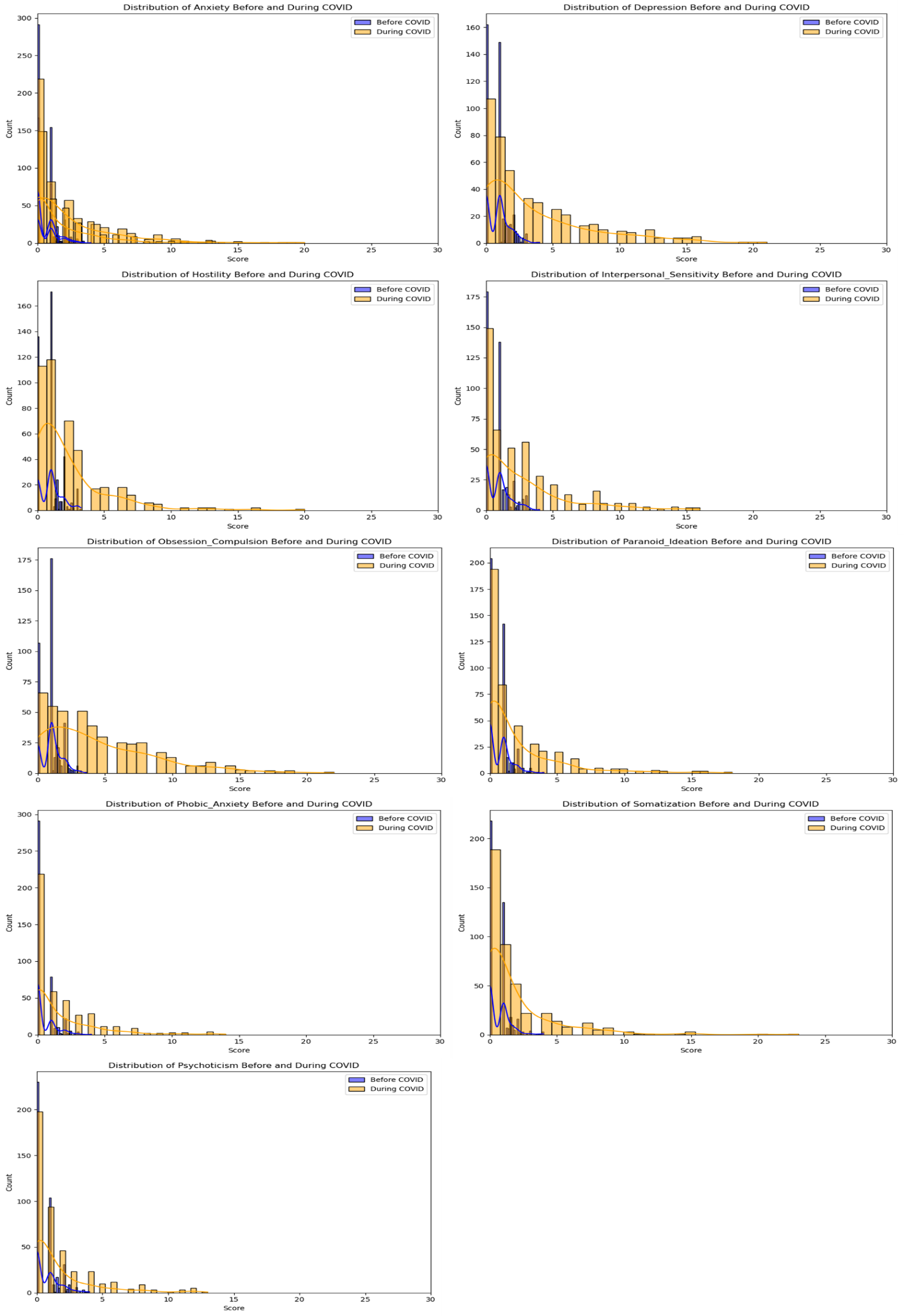
**

Supplementary Material 1 Distribution plots of BSI scales before and during COVID-19


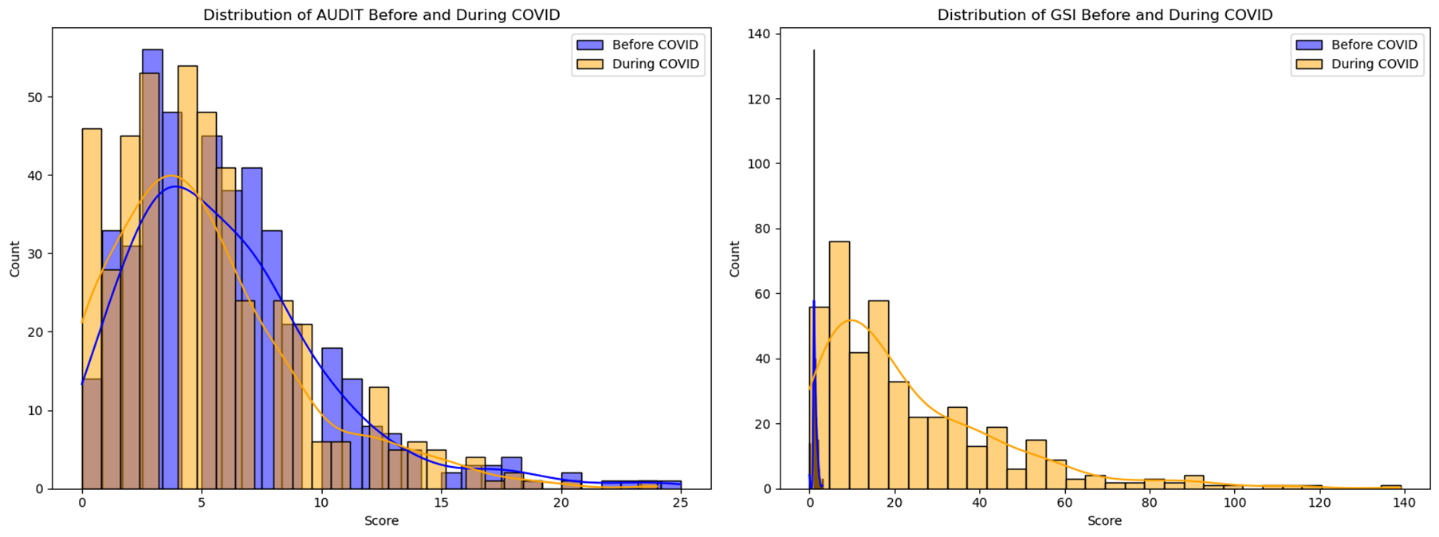


Supplementary Material 2 Distribution plots of BSI General Severity Index (GSI) and AUDIT Total score before and during COVID-19


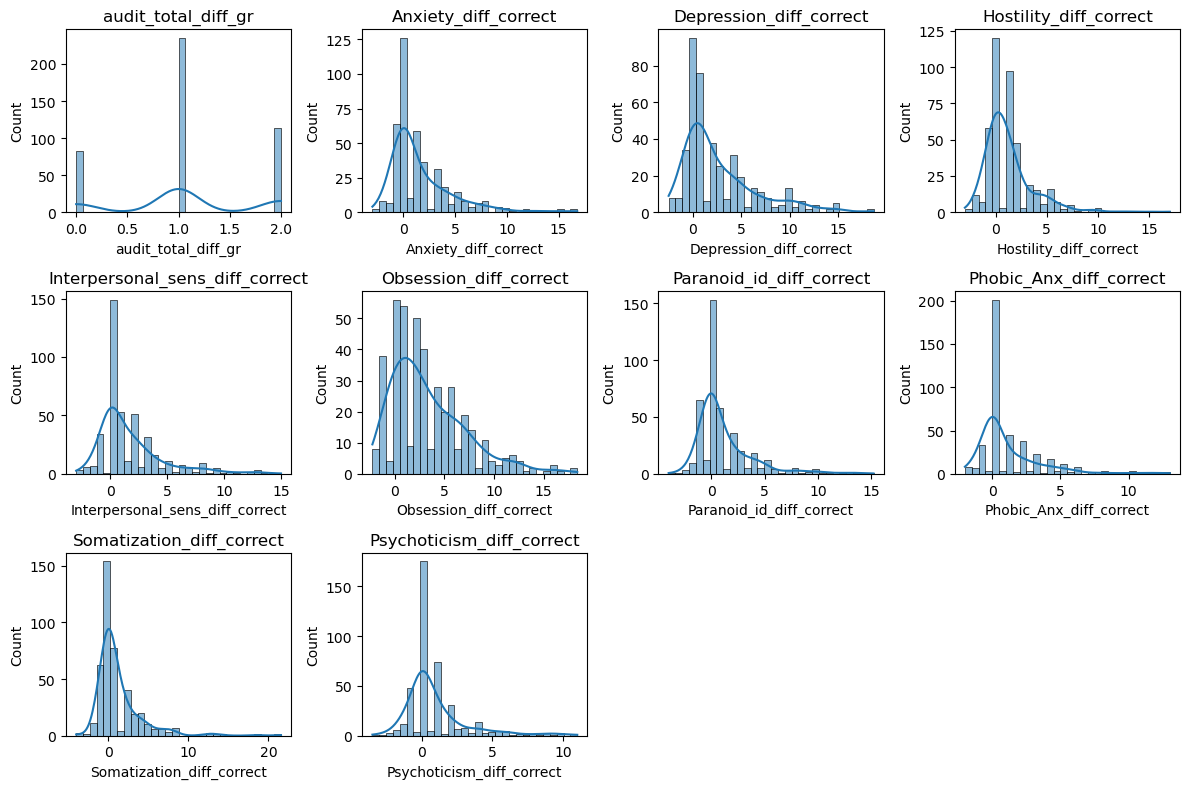


Supplementary Material 3 Distribution plots of BSI scales and AUDIT Total score for difference scores calculated for each participant by subtracting the pre-COVID-19 scores from the during-COVID-19

***Gender differences***

Interestingly, the results of the correlation analysis for the time before Covid-19 psychological distress (BSI) and alcohol consumption (AUDIT) scores showed notable differences between males and females. For females, significant positive correlations were found between AUDIT total scores and depression (*r* =.14, p < .001), hostility (*r* = .14, *p* < .001), interpersonal sensitivity (*r* = .12, *p* = .001), obsession-compulsion (*r* = .15, *p* < .001), paranoid ideation (*r* = .12, p = .001), phobic anxiety (*r* = .12, p =.001), somatization (*r* = .106, *p* = .005), psychoticism (*r* = .17, *p* < .001), and global severity index (*r* = .117, *p* = .002). For males, significant positive correlations were observed only between AUDIT total scores and psychoticism (r = .14, p < .001). Therefore, these results suggest that there are gender differences in the correlations between alcohol consumption and psychological distress indicators, with females generally exhibiting stronger correlations than males.


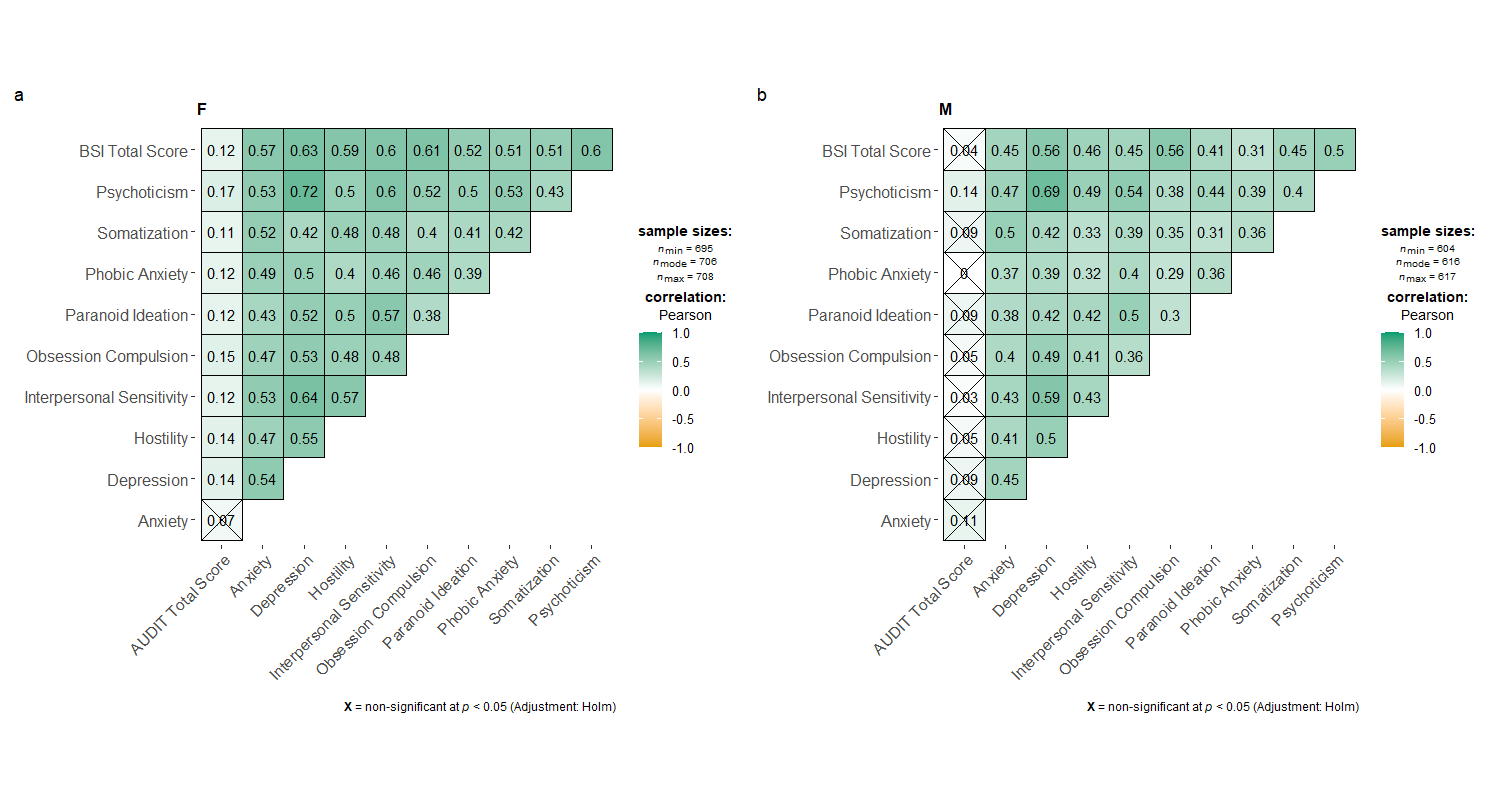


Supplementary Material 4 Correlation matrix for female (F) and male (M) group before Covid-19 (IMAGEN FU3)

Nevertheless, during Covid-19 the pattern changed, where for females, significant positive correlations were found only between AUDIT total scores and BSI global severity index (*r* = 0.182, *p* = 0.003). For males, no significant correlation was observed between AUDIT total scores and any of the BSI scales.


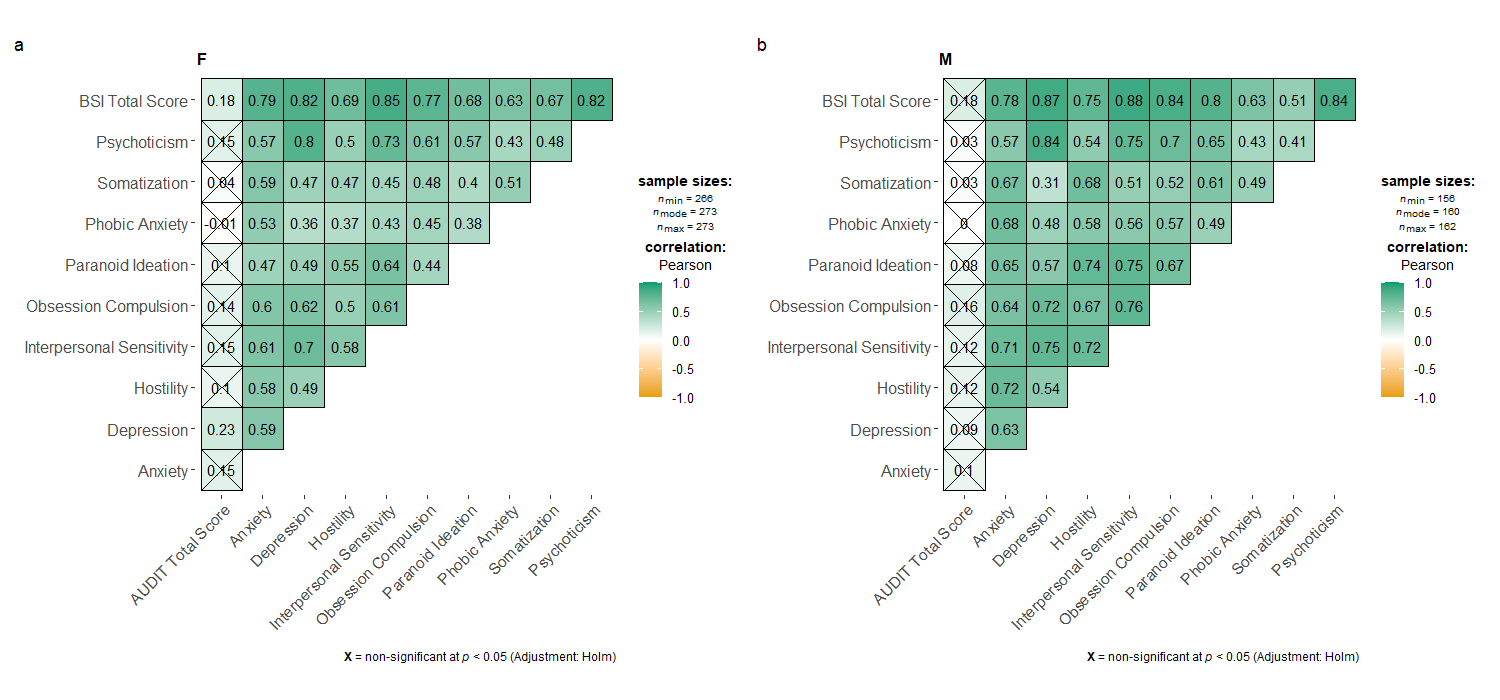


Supplementary Material 5 Correlation matrix for female (F) and male (M) group during Covid-19 (IMACOV BL)

These findings underscore the dynamic nature of the relationship between alcohol consumption and psychological distress, which may be influenced by external factors such as societal stressors, coping mechanisms, and changes in lifestyle patterns.

In the following step, changes in alcohol use and psychological distress scales were explored in both groups, where the paired samples t-tests were conducted for the female and male group separately. Based on the results of the paired samples t-test and effect size analysis, it's evident that significant changes occurred in the female group between the period before and during the Covid-19 pandemic. Firstly, in terms of alcohol consumption (AUDIT total scores), there was a statistically significant decrease from before (*M* = 5.28, *SD* = 4.093) to during Covid-19 (*M* = 3.70, *SD* = 3.830), with a mean difference of 1.583, t(128) = 6.21, p < .001, indicating a medium effect size (Cohen's d = 0.422). Secondly, concerning psychological distress indicators (BSI scales), significant increases were observed in depression, anxiety, hostility, interpersonal sensitivity, obsession-compulsion, paranoid ideation, phobic anxiety, somatization, psychoticism, and the global severity index. These changes were all statistically significant (p < .001) with medium to large effect sizes ranging from Cohen's d = -0.437 to -1.109.

In the male group, regarding alcohol consumption (AUDIT total scores), there was also a statistically significant decrease from before (*M* = 7.32, *SD* = 4.746) to during Covid-19 (M = 4.35, SD = 4.550), with a mean difference of 2.965, t(112) = 3.70, p < .001, indicating a large effect size (Cohen's d = 0.569). Similarly to the female group, concerning psychological distress indicators (BSI scales), significant increase were observed in anxiety, depression, hostility, interpersonal sensitivity, obsession-compulsion, paranoid ideation, phobic anxiety, somatization, and psychoticism, as well as the global severity index. All these changes were statistically significant (p < .001) with effect sizes ranging from medium to large, Cohen's d = -0.296 to -0.934.

Before Covid-19, AUDIT total scores were positively correlated with several dimensions of psychological distress, where significant positive correlation was observed between AUDIT total scores and depression (*r* = 0.103, *p* = 0.000), obsession-compulsion (*r* = 0.090, *p* = 0.001), paranoid ideation (*r* = 0.103, *p* < 0.01), somatization (*r* = 0.072, *p* < 0.01), psychoticism (*r* = 0.142, *p* < 0.01).

However, in contrast to the described results obtained for the time before Covid-19, AUDIT total scores remained positively correlated only with depression during Covid-19. Whereas, the strength of the correlation with depression increased compared to before COVID-19 (*r* = 0.142, *p* < 0.01).


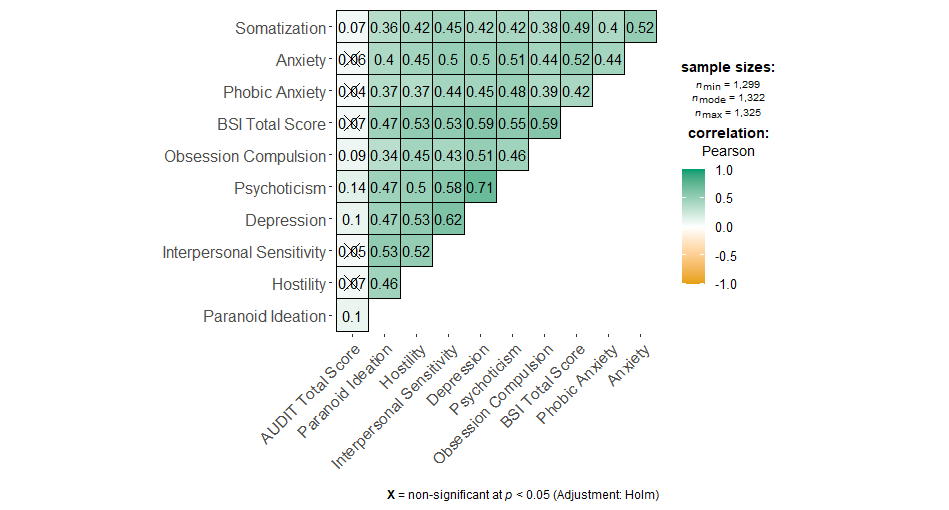


Supplementary Material 6 Correlation analyses between AUDIT and BSI for the whole sample before Covid-19


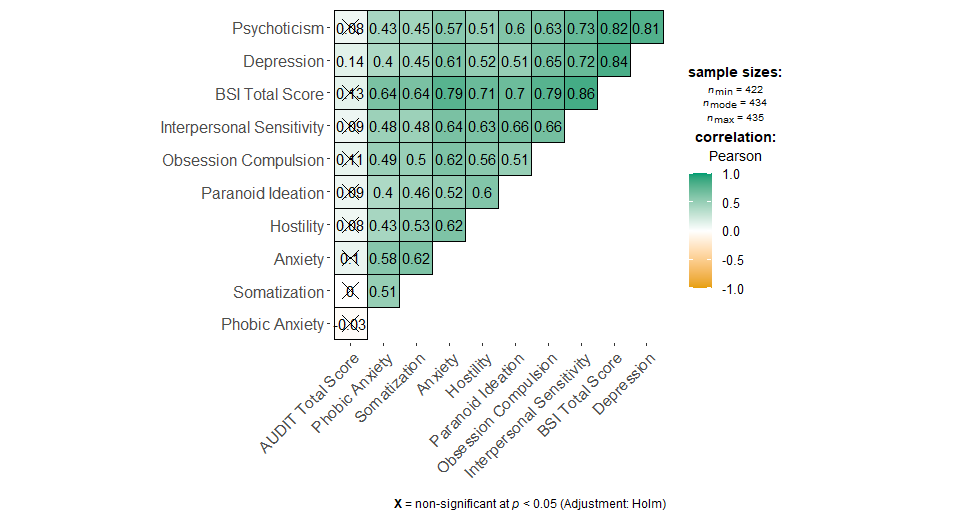


Supplementary Material 7 *Correlation analyses between AUDIT and BSI for the whole sample during Covid-19*

The comparison between the associations observed before and during COVID-19 revealed some valuable insights into how the relationship between alcohol consumption and psychological distress may have changed during the pandemic. Therefore, in the next step, paired samples t-tests were applied to investigate the differences in scores from before to during the COVID-19 crisis. However, due to the non-normal distribution and heterogeneity of variance in the data, the Yuen's trimmed means t-test was performed to ensure robust and reliable comparisons. The results indicate a significant increase in the total score of BSI (*t_Yuen_*(245) = -16.53, *p* = 0.00), depression (*t_Yuen_* (254) = -6.37, *p* = 0.00), interpersonal sensitivity (*t_Yuen_* (260) = -10.95, *p* = 0.00), and obsession-compulsion (*t_Yuen_* (260) = -10.95, *p* = 0.00) from before to during the COVID-19 crisis. Additionally, psychoticism showed a significant increase (*t_Yuen_* (257) = 3.34, *p* = 0.00) during this period. In contrast to these findings, there was a significant decrease in alcohol use.

Supplementary Material 8 Overview on the number of participants in each of clustered groups by BSI scales and alcohol use group.

|  | **Male (n=153)** | | | **Female (n=262)** | | |  |
| --- | --- | --- | --- | --- | --- | --- | --- |
|  | **persistent** | **increase** | **decrease** | **persistent** | **increase** | **decrease** | *p* |
| Alcohol consumption | 17,0 | 26,8 | 56,2 | 20,6 | 26,7 | 52,7 | .644 |
| Anxiety | 32,7 | 45,8 | 21,6 | 26,0 | 54,6 | 19,5 | .200 |
| Depression | 28,1 | 56,9 | 14,4 | 16,8 | 70,2 | 12,6 | **.012** |
| Hostility | 32,7 | 49,0 | 18,3 | 23,7 | 56,5 | 19,8 | .133 |
| Interpersonal Sensitivity | 44,4 | 41,8 | 13,7 | 27,1 | 61,5 | 11,1 | **.000** |
| Obsession Compulsion | 15,0 | 71,9 | 13,1 | 9,9 | 78,2 | 11,5 | .240 |
| Paranoid Ideation | 36,6 | 43,1 | 20,3 | 34,4 | 43,5 | 22,1 | .860 |
| Phobic Anxiety | 58,2 | 34,6 | 7,2 | 41,6 | 43,1 | 15,3 | **.002** |
| Somatization | 43,1 | 38,6 | 18,3 | 32,8 | 49,6 | 17,6 | .066 |
| Psychoticism | 52,3 | 32,0 | 15,7 | 34,0 | 46,6 | 19,5 | **.001** |
| BSI Total Score | 2,6 | 92,8 | 3,3 | 1,1 | 95,8 | 1,9 | .355 |

The further analysis of the data involved categorizing the participants into three groups based on their changes in various psychological distress indicators and alcohol consumption patterns before and during the Covid-19 pandemic. These groups were defined as follows: those whose values remained the same, those whose values increased, and those whose values decreased. Subsequently, the distribution of these groups was compared between males and females to discern any gender-specific trends.

The examination of alcohol consumption revealed no significant gender differences during the pandemic, with both males and females exhibiting similar proportions of individuals whose consumption remained the same, increased, or decreased. However, a gender disparity emerged when considering psychological distress indicators. In terms of depression, a notable gender difference was observed, with a higher proportion of males experiencing an increase in depressive symptoms compared to females during the pandemic. Interestingly, gender-specific responses were evident in other domains of psychological distress. Females exhibited a greater propensity for decreases in interpersonal sensitivity, phobic anxiety, and psychoticism compared to males during the pandemic.

| AUDIT | ,074 | ,037 | -,04 | ,023 | ,09 | ,036 | ,009 | ,018 | ,003 | ,136* | **--** |
| --- | --- | --- | --- | --- | --- | --- | --- | --- | --- | --- | --- |
| BSI (Total) | ,223** | ,270** | ,198** | ,233** | ,345** | ,142* | ,186** | ,135* | ,072 | **--** | -,043 |
| Psychoticism | ,256** | ,351** | ,209** | ,323** | ,173** | ,305** | ,215** | ,155** | **--** | ,217** | -,055 |
| Somatization | ,294** | ,173** | ,261** | ,173** | ,155** | ,190** | ,160** | **--** | ,127 | ,035 | -,045 |
| Phobic  Anxiety | ,279** | ,190** | ,146** | ,170** | ,125* | ,160** | **--** | ,086 | ,277** | ,165* | ,025 |
| Paranoid  Ideation | ,228** | ,274** | ,232** | ,282** | ,113* | **--** | ,146* | ,074 | ,301** | ,165* | ,043 |
| Obsession  Compulsion | ,308** | ,168** | ,088 | ,299** | **--** | ,282** | ,175* | -,06 | ,266** | ,352** | ,018 |
| Interpersonal Sensitivity | ,409** | ,340** | ,267** | **--** | ,165* | ,343** | ,166* | ,068 | ,423** | ,218** | ,023 |
| Hostility | ,312** | ,347** | **--** | ,243** | ,236** | ,249** | ,186* | ,215** | ,219** | ,109 | ,049 |
| Depression | ,383** | **--** | ,181* | ,312** | ,223** | ,238** | ,238** | ,036 | ,462** | ,315** | -,078 |
| Anxiety | **--** | ,233** | ,231** | ,288** | ,136 | ,300** | ,323** | ,206** | ,305** | ,217** | -,005 |
|  | Anxiety | Depression | Hostility | Interpersonal  Sensitivity | Obsession  Compulsion | Paranoid  Ideation | Phobic  Anxiety | Somatization | Psychoticism | BSI (Total) | AUDIT |

Supplementary Material 9 Kendal´s Tau Correlation

Note: Kendal´s Tau Correlation for grouped variables (increase – decrease – persistent) for females above the diagonal and for males below the diagonal line.** p<0.001; * p<0.05

**
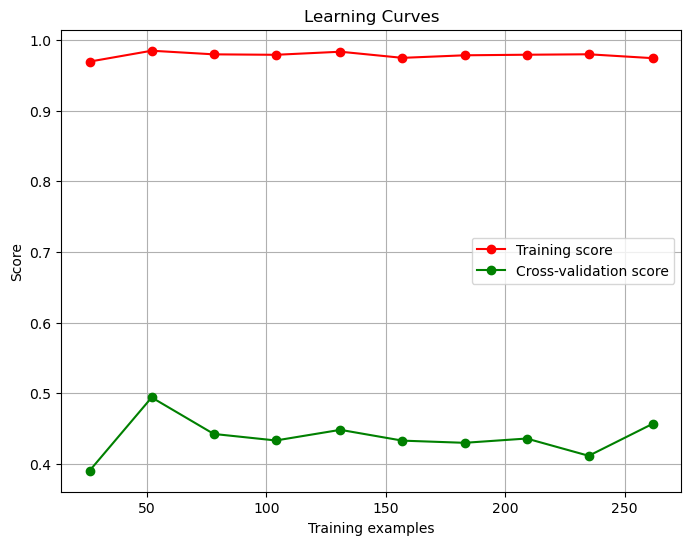
**

Supplementary Material 10 Learning curves for SHAP models

**
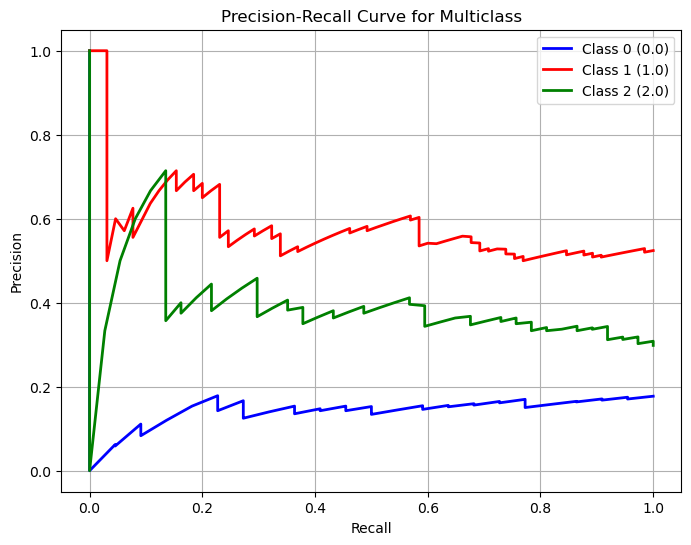
**

Supplementary Material 11 Precision-Recall Curve for multiclass of the classifier SHAP model; class 0 (no change in AUDIT scores), class 1 (decrease in AUDIT scores), and class 2 (increase in AUDIT scores),

**
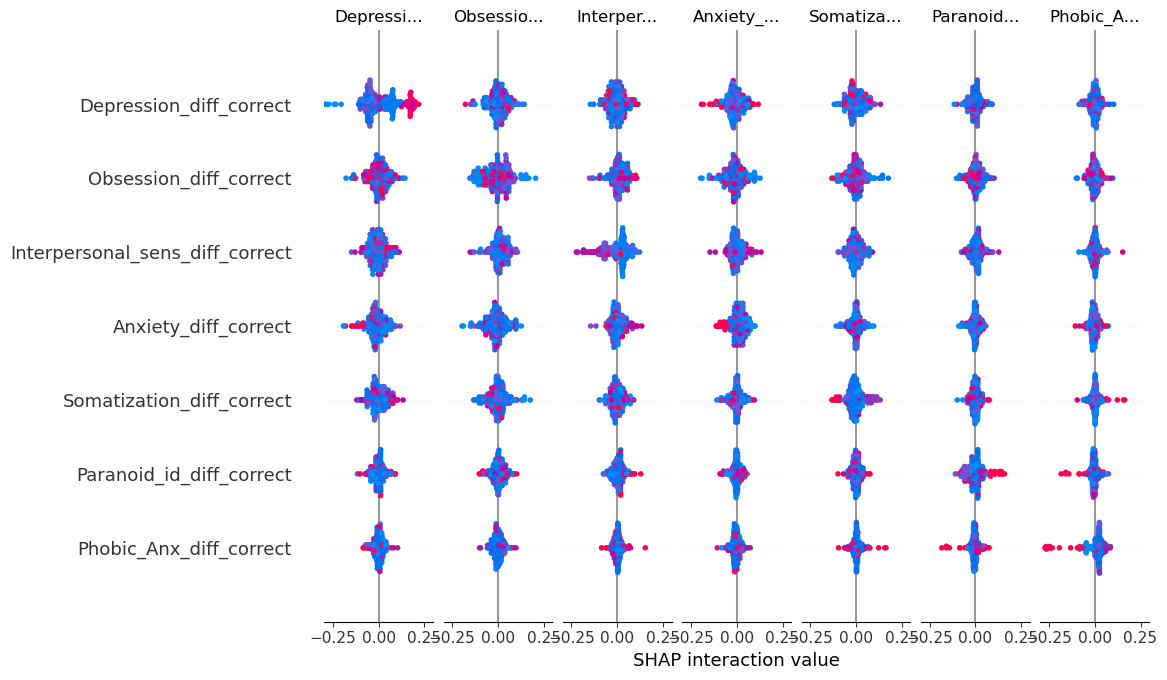
**

Supplementary Material 12 SHAP interactive plot across BSI subscales

**
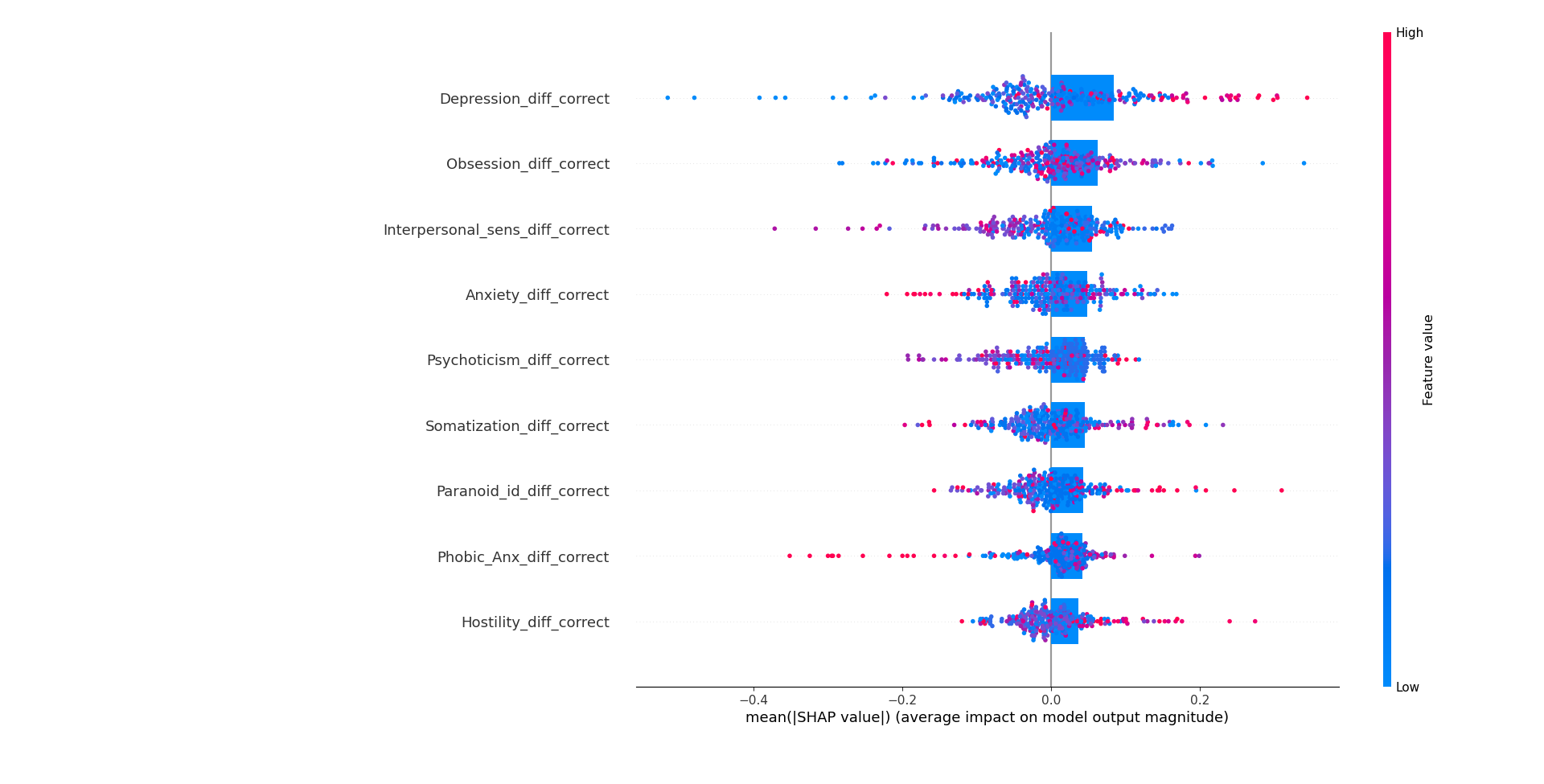
**

Supplementary Material 13 SHAP analysis results with change in alcohol use as a target variable and change in BSI scales as predictors


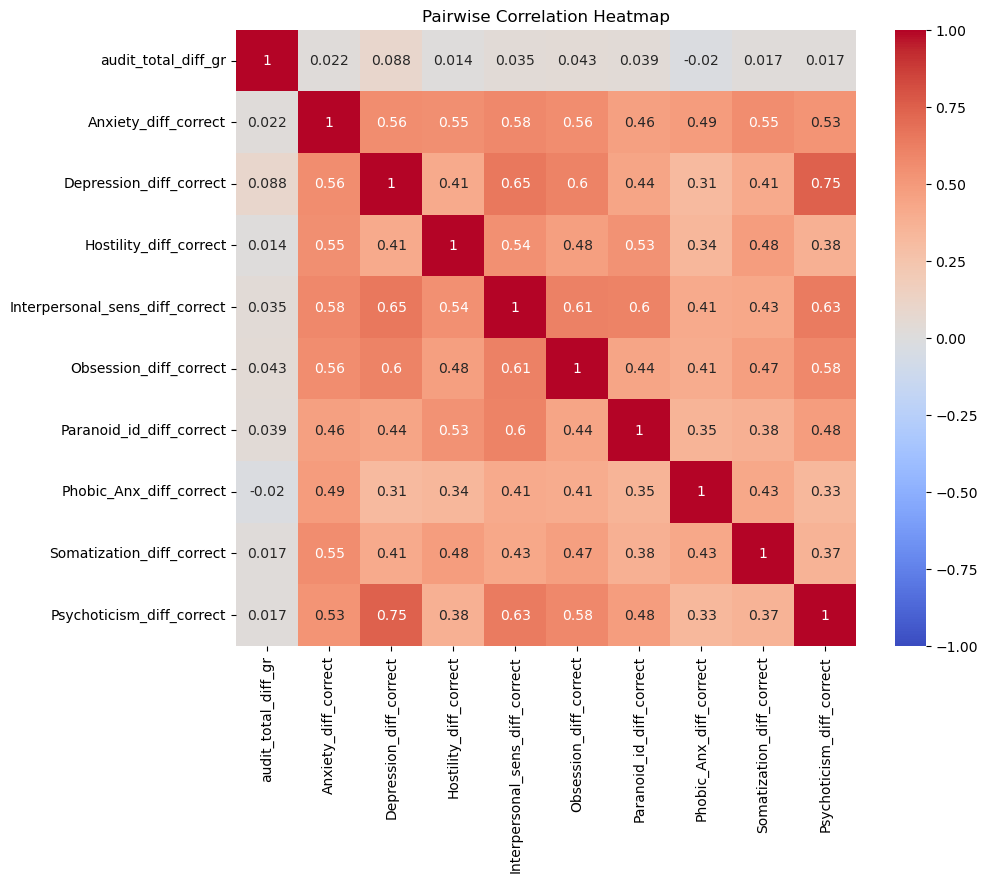


Supplementary Material 14 Correlation matrix of change scores by BSI scales and alcohol use

Supplementary Material 15 Partial correlation of BSI subscales and AUDITT Total score

|  | 1 | 2 | 3 | 4 | 5 | 6 | 7 | 8 | 9 | 10 |
| --- | --- | --- | --- | --- | --- | --- | --- | --- | --- | --- |
| 1. Anxiety (T1) |  |  |  |  |  |  |  |  |  |  |
| 2. Depression (T1) | .523** |  |  |  |  |  |  |  |  |  |
| 3. Hostility (T1) | .477** | .555** |  |  |  |  |  |  |  |  |
| 4. Interpersonal Sensitivity (T1) | .448** | .629** | .509** |  |  |  |  |  |  |  |
| 5. Obsession Compulsion (T1) | .428** | .518** | .437** | .439** |  |  |  |  |  |  |
| 6. Paranoid Ideation (T1) | .360** | .518** | .499** | .521** | .323** |  |  |  |  |  |
| 7. Phobic Anxietx (T1) | .461** | .464** | .388** | .423** | .368** | .345** |  |  |  |  |
| 8. Somatization (T1) | .511** | .412** | .432** | .383** | .332** | .320** | .397** |  |  |  |
| 9. Psychoticism (T1) | .492** | .727** | .501** | .564** | .435** | .440** | .449** | .412** |  |  |
| 10. Global Severity Index (T1) | .561** | .661** | .562** | .561** | .583** | .503** | .442** | .475** | .417** |  |
| 11. AUDIT (Total score, T2) | -.015 | .000 | -.024 | .019 | .072 | -.001 | -.040 | .013 | -.029 | .004 |

*Note*: ***p* < .001; included covariates: AUDIT Total score at T1, age and center
